# Role of Extracellular Carbonic Anhydrase in Dissolved Inorganic Carbon Uptake in Alkaliphilic Phototrophic Biofilm

**DOI:** 10.3389/fmicb.2018.02490

**Published:** 2018-10-22

**Authors:** Tong Li, Christine E. Sharp, Maryam Ataeian, Marc Strous, Dirk de Beer

**Affiliations:** ^1^Microsensor Group, Max-Planck-Insititute for Marine Microbiology, Bremen, Germany; ^2^Department of Geoscience, University of Calgary, Calgary, AB, Canada

**Keywords:** dissolved inorganic carbon, phototrophic biofilm, cyanobacteria, alkaliphilic biofilm, extracellular carbonic anhydrase, photosynthetic productivity

## Abstract

Alkaline Soda Lakes are extremely productive ecosystems, due to their high dissolved inorganic carbon (DIC) concentrations. Here, we studied the dynamics of the carbonate system, in particular, the role of extracellular carbonic anhydrase (eCA) of an alkaliphilic phototrophic biofilm composed of bacteria enriched from soda lake benthic mats. By using measurements with microsensors and membrane inlet mass spectrometry, combined with mathematical modeling, we show how eCA controls DIC uptake. In our experiments, the activity of eCA varied four-fold, and was controlled by the bicarbonate concentration during growth: a higher bicarbonate concentration led to lower eCA activity. Inhibition of eCA decreased both the net and the gross photosynthetic productivities of the investigated biofilms. After eCA inhibition, the efflux of carbon dioxide (CO_2_) from the biofilms increased two- to four-fold. This could be explained by the conversion of CO_2_, leaking from cyanobacterial cells, by eCA, to bicarbonate. Bicarbonate is then taken up again by the cyanobacteria. In suspensions, eCA reduced the CO_2_ leakage to the bulk medium from 90 to 50%. In biofilms cultivated at low bicarbonate concentration (~0.13 mM), the oxygen production was reduced by a similar ratio upon eCA inhibition. The role of eCA in intact biofilms was much less significant compared to biomass suspensions, as CO_2_ loss to the medium is reduced due to mass transfer resistance.

## Introduction

Alkaline Soda lakes are extremely productive due to the high DIC availability, and can be found in various geographical locations around the globe (Melack, 1981; Priscu et al., 1982; Kompantseva et al., 2009). Soda lakes are believed to have existed throughout the geological record of Earth, and are abundant in dry terrestrial biomes. These lakes support the growth of an large array of microorganisms that are of ecological and economic importance (Antony et al., 2013). Microalgae that thrive in the high pH and salinity can highly efficiently photosynthesize due to the elevated dissolved organic carbon (DIC) levels. This makes biofilms from alkaline environments potentially useful for carbon capture. The high medium pH can scrub effectively CO_2_ from the exhaust gasses from large CO_2_ producing industrial units, elevating the DIC in the medium to very high levels, while phototrophs can maintain the high pH in the scrubber liquid. Initial studies on the phototrophic microorganisms of natural alkaline and saline lakes, especially the biofilm forming cyanobacteria, demonstrated their very high phototrophic activity (Sharp et al., 2017). A mechanistic understanding of the DIC uptake in the biomass is desired and the aim of this study.

DIC exists in aqueous solution in 4 forms: as carbon dioxide (CO_2_), carbonic acid (H_2_CO_3_), bicarbonate ions (${\text{HCO}}_{3}^{-}$), and carbonate ions (${\text{CO}}_{3}^{2-}$).

(1) $$\begin{array}{l} CO_{2}\Leftrightarrow H_{2}CO_{3}\Leftrightarrow HCO_{3}^{-}\Leftrightarrow CO_{3}^{2-} \end{array}$$

The interconversion between CO_2_ and H_2_CO_3_ is relatively slow, with an 90% equilibration in 20 s under typical conditions, while the rest of the carbonate system equilibrates instantly. The highly effective enzyme carbonic anhydrase (CA) accelerates the reversible hydration of CO_2_. For convenience, the species CO_2_ and H_2_CO_3_ are often taken together and the hydration of CO_2_ is then noted to produce bicarbonate (${\text{HCO}}_{3}^{-}$) (Giordano et al., 2005).

Phototrophic organisms are able to take up both CO_2_ and ${\text{HCO}}_{3}^{-}$ as the carbon source for photosynthesis, whereby an essential difference is that CO_2_ can passively pass membranes and ${\text{HCO}}_{3}^{-}$ is actively taken up by transporters, driven by membrane potentials or ATP. Thus, uptake of ${\text{HCO}}_{3}^{-}$ can be controlled by the cell through adjusting the amount and affinity of the transporters. In photosynthesis, during carbon fixation, Ribulose-1,5-bisphosphate carboxylase/oxygenase (RuBisCo) only accepts CO_2_ as the substrate, and ${\text{HCO}}_{3}^{-}$ is first converted to CO_2_ (Giordano et al., 2005).

CA is crucial in CO_2_ exchanging transport tissue, e.g., in lungs, and also is essential in DIC uptake by phototrophic microorganisms. It plays an important but not yet entirely understood role in the carbon concentration mechanism (CCM). Intracellular carbonic anhydrase (iCA) facilitates rapid ${\text{HCO}}_{3}^{-}$ to CO_2_ conversion in the direct vicinity of RuBisCo, thus increasing the CO_2_ concentration around RuBisCo. The ensuing elevation of the CO_2_/O_2_ ratio increases its efficiency as carboxylase (Giordano et al., 2005). Extracellular carbonic anhydrase (eCA) has been detected in several phototrophic microorganisms, both in cyanobacteria and in eukaryotes, and is thought to have a role in DIC uptake into the cell (Katsunori and Shigetoh, 1986; Nimer et al., 1999; Kupriyanova et al., 2003, 2007, 2011; Karim et al., 2011; Hopkinson et al., 2013; Hamizah et al., 2017). This may be increasingly important at higher pH and/or lower dissolved free CO_2_ concentrations (i.e., hyperalkaline environments). So far, studies on the role of iCA and eCA in photosynthesis focused on phytoplankton rather than biofilms/mats (Katsunori and Shigetoh, 1986; Nimer et al., 1999; Kupriyanova et al., 2003, 2007, 2011; Karim et al., 2011; Hopkinson et al., 2013; Hamizah et al., 2017). Due to mass transfer resistances, the pH value in mats can be very high during photosynthesis, likely enhancing the role of eCA for DIC uptake and photosynthesis (Revsbech et al., 1981; Revsbech and Jørgensen, 1983; Revsbech, 1989; Jensen et al., 1993; Epping et al., 1999; Ionescu et al., 2014; Nielsen et al., 2015). Indeed, the mat-forming alkaliphilic cyanobacterium *Microcoleus chthonoplastes* possesses eCA, whose activity increases with pH values (Kupriyanova et al., 2003, 2007, 2011).

We aimed to investigate how eCA activity impacts DIC uptake and productivity. With organisms enriched from soda-lake benthic mats, an alkaliphilic phototrophic biofilm was created as a model system. The DIC uptake and the role of eCA in the DIC dynamics of this model system were investigated with a combination of methods, including microsensors, membrane inlet mass spectrometer, and mathematically modeling. We tested the hypothesis that the eCA is important for DIC uptake, especially at low bicarbonate concentrations. We expected that the cells will increase eCA activity when the DIC is limiting photosynthesis.

## Methods and materials

### Sample preparation

The biomass used for inoculating the investigated biofilm was prepared as described previously (Sharp et al., 2017). In short, phototrophic benthic mat samples were collected from four soda-lakes on the Cariboo Plateau, British Columbia in May 2015. Mat samples were homogenized and mixed in equal wet weight proportions. Phototrophs were enriched in flat panel photobioreactors (PBRs) with high pH and high alkalinity medium (“3e medium,” Table 1). The PBRs were exposed to 80 μmol photon m^−2^ s^−1^ red light (fluorescent lamp equipped with a red-pass filter, Congo Red) under a 16:8 light/dark cycle at 25 ± 2°C. During cultivation, the medium was exchanged regularly and the biomass in the PBR was harvested once every week. The enriched biomass was dominated by the cyanobacterium *Phormidium kuetzingianum* (>50% relative abundance) (Sharp et al., 2017).

**Table 1:** Composition of the 3e medium.

**Chemical**	**Final concentration**
^b^NaHCO_3_	85 g·L^−1^
K_2_HPO_4_	1 g·L^−1^
MgSO_4_·7H_2_O	246 mg·L^−1^
NH_4_Cl	218 mg·L^−1^
Na_3_EDTA	500 μg·L^−1^
FeSO_4_·7H_2_O	200 μg·L^−1^
ZnSO_4_·7H_2_O	10 μg·L^−1^
MnCl_2_·4H_2_O	3 μg·L^−1^
H_3_BO_3_	30 μg·L^−1^
CoCl_2_·6H_2_O	20 μg·L^−1^
CuCl_2_·2H_2_O	1 μg·L^−1^
NiCl_2_·6H_2_O	2 μg·L^−1^
Na_2_MoO_4_·2H_2_O	3 μg·L^−1^

*If required, buffered with 20mM Tris buffer; ^b^If required, DIC concentration adjusted by substituting NaHCO_3_ with NaCl.

Biomass was harvested from the photobioreactors, resuspended in medium, allowed to settle for 1 h, after which the supernatant was replaced with DIC free medium and resuspended. After 5 min, the biomass was concentrated by centrifugation (500 × G, 1 min), and the pellet resuspended in DIC free medium. This process was repeated twice. Agar plates (4% and amended with corresponding media) were submerged in “3e medium” modified to have 0, 0.01, 01, 0.5, or 1 M DIC. Then the concentrated biomass was inoculated onto agar plates. Biofilm formation on the agar plate occurred within 12 h. The DIC concentration was adjusted by decreasing the NaHCO_3_ concentration of the “3e medium,” compensated by NaCl to keep Na^+^ constant. Due to equilibration with air, 0 M DIC actually contained about 0.13 mM DIC. Solidified agar plates each with a surface area of 23 cm^2^ were used as substrate for biofilm support. For every DIC concentration, 2 such agar plates were inoculated, and cultivated in their respective media using halogen lamps (General Electric, USA) with continuous illumination and a light intensity of 100 μmole·m^−2^·s^−1^ at 23°C, submerged in a flow cell (2 cm under surface). Each flow cell system had a total volume of 1.5 L, including the recirculation reservoir. The agar plates were cultivated for 5–7 days without changing the medium before measurement, the medium was circulated using a peristatic pump with a flow rate of 0.65 mL·min^−1^.

### Microsensor measurements

Profiles of oxygen (O_2_) concentration (Revsbech, 1989) and pH (Jensen et al., 1993) were measured using microsensors with tip diameter of maximally 15 μm. The LIX pH sensors were prepared with protective protein coating (De Beer et al., 1997). Gross photosynthetic productivity was measured by the light/dark shift method using an oxygen microsensor with <0.2 s response time (Revsbech et al., 1981; Revsbech and Jørgensen, 1983). The microsensors were prepared, calibrated and used as described previously (Revsbech et al., 1981; Revsbech and Jørgensen, 1983; Revsbech, 1989; Jensen et al., 1993; Epping et al., 1999). The agar plate with the biofilm was place in 300 mL of freshly prepared medium (with identical composition as during cultivation). The water column above the biofilm surface was 2 cm. The sensors were positioned perpendicularly to the biofilm surface. The sample was equilibrated for 10 min before the first microsensor measurement. The medium was mixed by an airstream across the medium surface. The measurements were first performed on biofilms without inhibitor. Then, an eCA inhibitor, acetazolamide (AZ) (Mercado et al., 2009; Hopkinson et al., 2013) was injected 3–4 min before the measurement into the measurement chamber (final concentration of 150 μM). During microsensor measurements, the illumination was identical to that applied during biofilm cultivation. Calibration of the oxygen sensors was performed using N_2_ and air bubbled “3e medium,” the O_2_ concentration of air saturated medium was calculated using the ambient temperature and salinity of the respective media (Sherwood et al., 1991). Calibration of the pH sensors was performed using commercial buffer solutions (pH 7 and 9, Fluka, Germany).

### Membrane inlet mass spectrometry (MIMS)

MIMS measurements were carried out using an Agilent 5977A MSD (Agilent, USA) mass spectrometer connected to a custom-made membrane inlet cuvette, the construction of the cuvette has been described previously (Rost et al., 2007; Beckmann et al., 2009). A PTFE membrane (10 μm thick, 0.33 cm^2^ surface area; Reichelt Chemical Technique, Germany) was selected as the inlet. The 6 mL cuvette had an injection port at the top of the cuvette. A suspended magnetic stirrer was positioned directly above the membrane, and the temperature of the cuvette was kept constant with a build-in water jacket at 20°C. Biomass was harvested from the agar plates, washed twice in DIC free medium, and consequently concentrated by centrifugation (1,000 × G, 30 s), then homogenized by forcing the biomass through a 0.2 mm inner diameter syringe needle several times. This procedure broke the biofilm into small and relatively uniformly sized pieces (ca. 0.1 mm in diameter). Microscopic inspection of the concentrated biomass and the absence of pigments in the supernatant showed that the procedure does not disrupt cells. To determine the ash free dry weight (AFDW) of the biomass, the biomass was first washed twice with DIC free medium, then dried at 70°C overnight. The AFDW was calculated as the weight loss after combustion at 550°C for 3 h.

The eCA activity was measured with a method as described previously (Palmqvist et al., 1994). The MIMS cuvette was covered to exclude all light. DIC solution, prepared using NaH^13^C^18^O_3_, was injected into the MIMS cuvette containing DIC free medium buffered to pH 8.7 (Tris buffer) to achieve a final DIC concentration of 1 mM. The log enrichment of ^13^C^18^O_2_ was then monitored for at least 10 min. The log-enrichment of ^13^C^18^O_2_ was calculated as:

(2) logenrichmntC13o218=logICC13o218×100ICC13o218+ICC13O18O16++ICC13o216,

*IC* with the different subscripts are the mass spectrometer counts of the different ^13^CO_2_ species.

**Figure 1:**
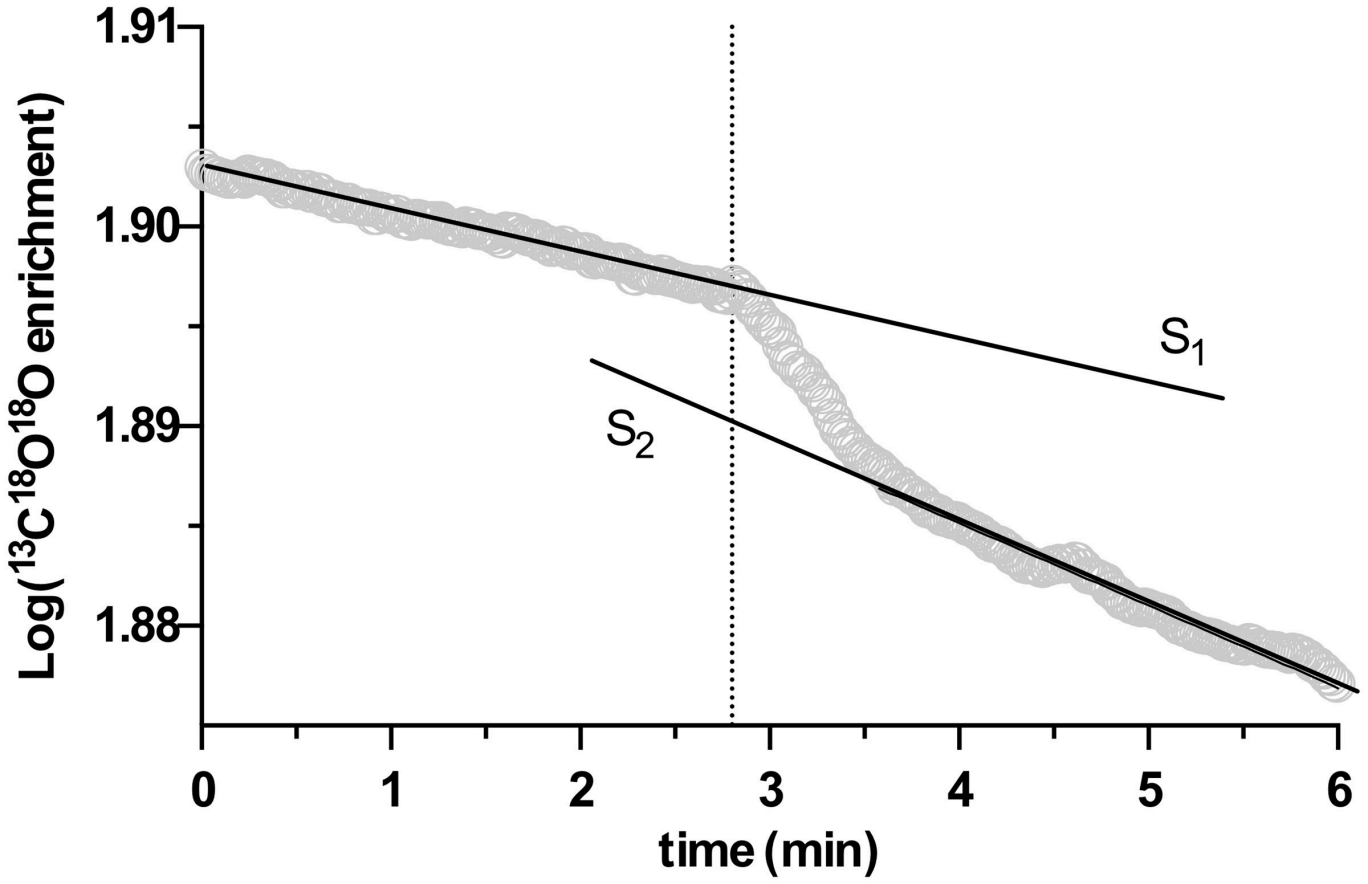
An example of extracellular carbonic anhydrase (eCA) activity measurement with membrane inlet mass spectrometry (MIMS), performed in the dark. X-axis is time, Y-axis represents the log enrichment of ^13^C^18^O^18^O, calculated with Equation (1). At the time point indicated by the dotted line, biomass was injected. S_1_ and S_2_ depict the slopes of linear regressions performed on the rate of decay of log enrichment of ^13^C^18^O^18^O before and after biomass injection.

Subsequently, 0.6 mL of the processed biomass was injected into the cuvette suspension (corresponding to a final biomass AFDW concentration between 0.18 and 0.26 mg·mL^−1^ in the cuvette), and the change of the log-enrichment of ^13^C^18^O_2_ was monitored for a further 10 min. Due to its catalytic activity, eCA will increase the ^18^O lost rate of ^13^C^18^O_2_, thus accelerate the decrease of the log enrichment of ^13^C^18^O_2_. Consequently, eCA activity can be quantified as the change in the decay rate of the log-enrichment of ^13^C^18^O_2_ per g AFDW (Palmqvist et al., 1994). An example of the acquired time vs. log enrichment chart is given in Figure 1, and the eCA activity was quantified as:

(3) $$\begin{array}{l} activity_{eCA}=\frac{S_{2}-S_{1}}{S_{1}}\cdot \frac{1}{AFDW}, \end{array}$$

in Equation (3), *S*_1_ and *S*_2_ are the slope of the log-enrichment of ^13^C^18^O_2_ change before and after injection of biomass, respectively, as shown in Figure 1, and *AFDW* is the ash-free dry weight of the biomass injected in this particular measurement.

The MIMS setup was also applied to investigate DIC uptake of the biomass harvested from the agar plates: The MIMS cuvette was filled with “3e medium” modified with 0.1 M DI^13^C prepared using NaH^13^CO_3_ and buffered to pH 8.7 using Tris buffer. To initiate the measurement, DIC free medium washed and homogenized biomass was injected in the dark into the cuvette (final AFDW concentration between 0.18 and 0.26 mg·mL^−1^). After 3 min, 100 μmole·m^−2^·s^−1^ light was supplied by turning on a halogen lamp. The signals of O_2_, ^12^CO_2_, and ^13^CO_2_ (m/z value 32, 44, 45, respectively) were monitored. After the steady state was reached (indicated by linear behavior of the signals, achieved within 5 min), the signals were monitored for at least 5 more minutes to acquire steady state rates (linear phase slope). Then, an eCA inhibitor, dextran-bound-acetazolamide (DBAZ) was added into the cuvette to a final concentration of 150 μM (Hopkinson et al., 2013). As DBAZ cannot enter the cell, it inhibits only eCA. The aforementioned signals were monitored further for at least 10 min, and the linear slopes were calculated. Finally, to acquire dark respiration rates, the light was turned off for the last 10 min of the measurement. To calculate the absolute rate, the MIMS signals were calibrated using linear calibration curves (for O_2_, N_2_ and air bubbled “e3 medium”; for ^13^CO_2_, N_2_ bubbled DIC free “3e medium,” 0.001 M and 0.1 M DI^13^C “e3 medium,” buffered to pH 8.7). Also, the consumption of O_2_ by the MIMS, and the effect of temperature and salinity on O_2_ concertation were corrected (Sherwood et al., 1991). The equilibrium constants, *K*_1_ and *K*_2_, of the carbonate system for calculating various DIC concentrations was given in **Table 3** (Davies, 1962; Eigen, 1964; Steiner et al., 1975; Johnson, 1982; DOE, 1994; Millero et al., 2002; Schulz et al., 2006; Wolf et al., 2007; Lee and Rasaiah, 2013). If not otherwise specified, all curves in the figures were created using one phase log-association provided by the software Prism (Graphpad, USA).

### Mathematical model

**Figure 2:**
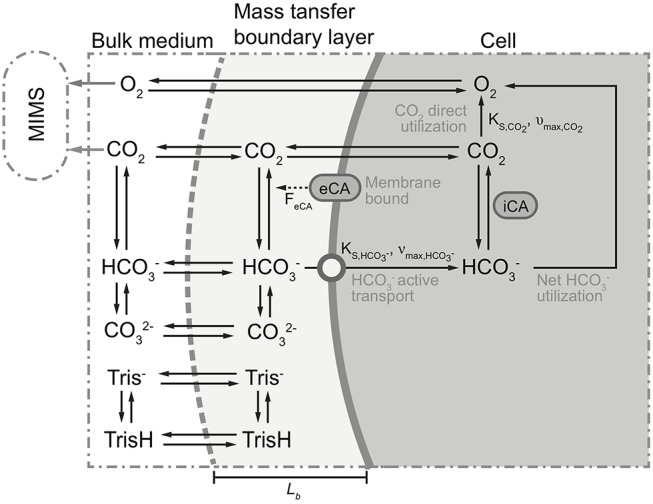
Schematic of the proposed model. Black solid arrows indicate possible directions of transfer and/or conversion. For some processes, parameters controlling these processes are given parallel to (i.e., next to) the arrows. Dotted arrow indicates participation of eCA as catalyst in the conversion process. Gray solid arrows show the concentrations measured by the membrane inlet mass spectrometry (MIMS). Top/bottom boundaries of the proposed model are marked with gray dashed line and thick solid gray line, respectively. The modeled compartments are marked at the top of the figure. For clarity, ^13^C was simplified as C, and H^+^ and OH^−^ ions are omitted. For more detailed description of the processes and parameters illustrated here, refer to Tables 2, 3.

A mathematical model was constructed to analyze the observed trends in the MIMS measurements. The model was based on the concept of Schulz et al. (2006), modified to incorporate biological processes (Figure 2). In short, the dynamic model calculates fluxes in and out of cells, based on mass balance equations, taking into account diffusion, chemical processes (e.g., acid-base reaction), biological processes (e.g., CO_2_ fixation). As we considered only the fate of added ^13^CO_2_ label, dark respiration was not included. The equations describing the considered processes are given in Table 2. The values of the used parameters are given in Table 3. The biological processes were considered to occur only in the cells, and eCA was considered to be cell-bound only. The DIC dynamics are calculated in the bulk liquid, i.e., the free-flowing medium in the MIMS measurement chamber, using the biomass conversion rates and fluxes between biomass and medium. The boundaries were set to be the cell surface and the interface between the flow boundary layer and the bulk liquid, respectively. Initial concentrations of the dissolved species were set to the same values as in air saturated, freshly prepared “3e medium” with 0.1 M DI^13^C, buffered to pH 8.7 with 20 mM Tris. The effect of eCA was modeled as pH dependent enhancement factors, *F*_*eCA*_, for reaction rates of CO_2_ hydration and ${\text{HCO}}_{3}^{-}$ dehydration (i.e., multiplied to the reaction rate constant) (Coleman, 2000; Supuran, 2016). This pH dependent enhancement factor is controlled by an imposed pH independent factor, *F*_*eCA*_. Higher *F*_*eCA*_ values represent stronger eCA activity, *F*_*eCA*_ = 1 indicates no eCA activity. eCA inhibition was simulated as reducing the reaction rate of $CO_{2}{+H}_{2}O⇌HCO_{3}^{-}+H^{+}$ to un-catalyzed values (i.e., set *F*_*eCA*_ to 1, refer to Tables 2, 3 and Figure 2). The model, which was a dynamic model represented by a combination of partial differential equations (Table 3), was solved by first converting the partial differential equations to a system of ordinary differential equations (ODEs), using the method of lines (Wouwer et al., 2014). The ODEs were then solved with the *ode15s* solver provided in the MATLAB software (version 2015b, MathWorks, USA). A parameter analysis of the model was performed by varying the values of model different parameters (refer to Table 3 and Figure 2).

**Table 2:** State variables and their corresponding expressions in the proposed model.

**State variable**	**Diffusion**	^**^Chemical reaction	^***^Biological process
$\frac{\partial [O_{2}]}{\partial t}=$	$-D_{O_{2}}\cdot \frac{d^{2}[O_{2}]}{dx^{2}}$	/	$\begin{array}{l} +\upsilon_{\max,CO_{2}}\cdot \frac{\left[CO_{2}\right]}{[CO_{2}]+K_{S,CO_{2}}}\\ +\nu_{max,HCO_{3}^{-}}\cdot \frac{\left[HCO_{3}^{-}\right]}{[HCO_{3}^{-}]+K_{S,HCO_{3}^{-}}}\cdot \Upsilon_{HCO_{3}^{-},O_{2}} \end{array}$
$\frac{\partial [CO_{2}]}{\partial t}=$	$-D_{CO_{2}}\cdot \frac{d^{2}[CO_{2}]}{dx^{2}}$	$\begin{array}{l} +\left(F_{eCA}\cdot k_{-1}\cdot [H^{+}\right]+k_{-2})\cdot [HCO_{3}^{-}]\\ -(F_{eCA}\cdot k_{+1}+k_{+2}\cdot [OH^{-}])\cdot [CO_{2}] \end{array}$	$\begin{array}{l} -\upsilon_{\max,CO_{2}}\cdot \frac{\left[CO_{2}\right]}{[CO_{2}]+K_{S,CO_{2}}}\\ +\nu_{max,HCO_{3}^{-}}\cdot \frac{\left[HCO_{3}^{-}\right]}{[HCO_{3}^{-}]+K_{S,HCO_{3}^{-}}}\cdot (1-\\ \Upsilon_{HCO_{3}^{-},O_{2}}) \end{array}$
$\frac{\partial \left[HCO_{3}^{-}\right]}{\partial t}=$	$-D_{HCO_{3}^{-}}\cdot \frac{d^{2}\left[HCO_{3}^{-}\right]}{dx^{2}}$	$\begin{array}{l} -\left(F_{eCA}\cdot k_{-1}\cdot [H^{+}\right]+k_{-2})\cdot [HCO_{3}^{-}]\\ +(F_{eCA}\cdot k_{+1}+k_{+2}\cdot [OH^{-}])\cdot [CO_{2}]\\ -(k_{-3}^{H^{+}}+k_{+3}^{OH^{-}}\cdot [OH^{-}])\cdot [HCO_{3}^{-}]\\ +(k_{+3}^{H^{+}}\cdot [H^{+}]+k_{-3}^{OH^{-}})\cdot [CO_{3}^{2-}] \end{array}$	$-\nu_{max,HCO_{3}^{-}}\cdot \frac{\left[HCO_{3}^{-}\right]}{\left[HCO_{3}^{-}\right]+K_{S,HCO_{3}^{-}}}$
$\frac{\partial [CO_{3}^{2-}]}{\partial t}=$	$-D_{CO_{3}^{2-}}\cdot \frac{d^{2}[CO_{3}^{2-}]}{dx^{2}}$	$\begin{array}{l} +(k_{-3}^{H^{+}}+k_{+3}^{OH^{-}}\cdot [OH^{-}])\cdot [HCO_{3}^{-}]\\ -(k_{+3}^{H^{+}}\cdot [H^{+}]+k_{-3}^{OH^{-}})\cdot [CO_{3}^{2-}] \end{array}$	/
$\frac{\partial \left[TrisH\right]}{\partial t}=$	$-D_{Tris}\cdot \frac{d^{2}\left[TrisH\right]}{dx^{2}}$	$-k_{+5}\cdot \left[TrisH\right]+k_{-5}\cdot [H^{+}]\cdot \;[Tris^{-}]$	/
$\frac{\partial \left[Tris^{-}\right]}{\partial t}=$	$-D_{Tris}\cdot \frac{d^{2}\left[Tris^{-}\right]}{dx^{2}}$	$+k_{+5}\cdot [TrisH]-k_{-5}\cdot [H^{+}]\cdot \;[Tris^{-}]$	/
$\frac{\partial [H^{+}]}{\partial t}=$	$-D_{H^{+}}\cdot \frac{d^{2}[H^{+}]}{dx^{2}}$	$\begin{array}{l} +F_{eCA}\cdot k_{+1}\cdot [CO_{2}]\\ -F_{eCA}\cdot k_{-1}\cdot [H^{+}]\;\cdot [HCO_{3}^{-}]\\ +k_{-3}^{H^{+}}\cdot \left[HCO_{3}^{-}\right]-k_{+3}^{H^{+}}\cdot [H^{+}]\;\cdot [CO_{3}^{2-}]\\ +k_{+4}+k_{-4}\cdot [H^{+}]\;\cdot [OH^{-}]\\ +k_{+5}\cdot [TrisH]-k_{-5}\cdot [H^{+}]\cdot \;[Tris^{-}] \end{array}$	/
$\frac{\partial \left[OH^{-}\right]}{\partial t}=$	$-D_{OH^{-}}\cdot \frac{d^{2}\left[OH^{-}\right]}{dx^{2}}$	$\begin{array}{l} +k_{-2}\cdot [HCO_{3}^{-}]-k_{+2}\cdot [OH^{-}]\cdot [CO_{2}]\\ -k_{+3}^{OH^{-}}\cdot [OH^{-}]\cdot [HCO_{3}^{-}]+k_{-3}^{OH^{-}}\cdot [CO_{3}^{2}-]\\ +k_{+4}+k_{-4}\cdot [H+]\;\cdot [OH^{-}] \end{array}$	/

^*^Refer to Table 3 and Figure 2;

** *F_eCA_ equals 1 except at the surface of the cell and when no DBAZ is present; equals 10^12^ for compartment inside the cell to simulate the effect of iCA*.

*** *Only occurs in biomass (i.e., modeled as sink/source at the surface of the biomass clump)*.

**Table 3:** Parameters, their definitions, source and values as applied in the proposed model.

**Parameter**	**Definition**	**Source/Expression**	**References**	**Value**	**Unit**
*S*	Salinity	Calculated based on medium formula	/	61	‰
*T*	Temperature	Measured	/	293.15	K
*K*_1_	Equilibrium constant for H_2_CO_3_ dissociation	$10^{(}{\;}^{8.712+9.46\times 10^{-3}\cdot S-8.56\times 10^{-5}}\cdot S^{2}-\frac{1355.1}{T}$ ^−1.7976·ln(*T*))^	Millero et al., 2002	10^−5.864^	M
*K*_2_	Equilibrium constant for ${\text{HCO}}_{3}^{-}$ dissociation	$\begin{array}{l} 10{(}^{-17.001+0.01259\cdot S+7.9334\times 10^{-5}}\cdot S^{2}-\frac{936.291}{T}\\ +1.87354\cdot \ln(T)+2.61471\cdot \frac{S}{T}-0.07479\frac{S^{2}}{T}) \end{array}$	Millero et al., 2002	10^−8.893^	M
*K*_*w*_	Solubility product of water	/	DOE, 1994	10^−13.5^	M^2^
*K*_*Tris*_	Equilibrium constant for Tris deprotonation	Product data sheet (Sigma), ionic strength corrected	Johnson, 1982	10^−8.1^	M
*k*_+1_	Rate constant: $CO_{2}{+H}_{2}O\begin{array}{c} k_{+1}\\ ⇌\\ k_{-1} \end{array}HCO_{3}^{-}+H^{+}$	$e^{1246.98-\frac{6.19\times 10^{4}}{T}-183\cdot \ln\left(T\right)}$	Schulz et al., 2006	0.0238	s^−1^
*k*_−1_		*k*_+1_/*K*_1_	Eigen, 1964	1.74 × 10^4^	M^−1^·s^−1^
*k*_+2_	Rate constant: $CO_{2}+OH^{-}\begin{array}{c} k_{+2}\\ ⇌\\ k_{-2} \end{array}HCO_{3}^{-}$	$A\cdot e^{-\frac{90166.83}{R\cdot T}}/K_{w}$	^**^	6.74 × 10^3^	M^−1^·s^−1^
*k*_−2_		*k*_+2_·*K*_*w*_/*K*_1_	Schulz et al., 2006	1.56 × 10^−4^	s^−1^
$k_{+3}^{H^{+}}$	Rate constant: $CO_{3}^{2-}+H^{+}\begin{array}{c} k_{+3}^{H^{+}}\\ ⇌\\ k_{-3}^{H^{+}} \end{array}HCO_{3}^{-}$	/	Eigen, 1964	5.00 × 10^10^	M^−1^·s^−1^
$k_{-3}^{H^{+}}$		$k_{+3}^{H^{+}}\cdot K_{2}$	Schulz et al., 2006	63.96	s^−1^
$k_{+3}^{OH^{-}}$	Rate constant: $HCO_{3}^{-}+OH^{-}\begin{array}{c} k_{+3}^{OH^{-}}\\ ⇌\\ k_{-3}^{OH^{-}} \end{array}CO_{3}^{2-}{+H}_{2}O$	/	Eigen, 1964	6.00 × 10^9^	M^−1^·s^−1^
$k_{-3}^{OH^{-}}$		$k_{+3}^{OH^{-}}\cdot K_{w}/K_{2}$	Schulz et al., 2006	1.48 × 10^5^	s^−1^
*k*_+4_	Rate constant: $H_{2}O\begin{array}{c} k_{+4}\\ ⇌\\ k_{-4} \end{array}H^{+}+OH^{-}$	Eigen (1964)	Eigen, 1964	0.014	M·s^−1^
*k*_−4_		*k*_+4_/*K*_*w*_	Schulz et al., 2006	4.43 × 10^10^	M^−1^·s^−1^
*k*_+5_	Rate constant: $TrisH\begin{array}{c} k_{+5}\\ ⇌\\ k_{-5} \end{array}Tris^{-}+H^{+}$	*k*_+5_·*K*_*Tris*_	Schulz et al., 2006	3.16 × 10^3^	s^−1^
*k*_−5_		${10\cdot k}_{-3}^{H^{+}}$	Schulz et al., 2006	5.00 × 10^11^	M^−1^·s^−1^
υ_max_, *CO*__2__	Maximum CO_2_ utilization/uptake rate	Artificial value	/	5 × 10^−4^	M·s^−1^
*K*_*S, C*_*O*__2__	Half saturation constant for CO_2_ utilization/uptake	Artificial value	/	10^−3^	M
$\nu_{max,HCO_{3}^{-}}$	Maximum ${\text{HCO}}_{3}^{-}$ utilization/uptake rate	Artificial value	/	5 × 10^−4^	M·s^−1^
$K_{S,HCO_{3}^{-}}$	Half saturation constant for ${\text{HCO}}_{3}^{-}$ utilization/uptake	Artificial value	/	10^−3^	M
$\Upsilon_{HCO_{3}^{-},O_{2}}$	Net ${\text{HCO}}_{3}^{-}$ bicarbonate utilization rate	Artificial value	/	0.6	/
$F_{eCA}^{*}$	pH independent eCA enhancement factor	Artificial value	^***^	100	/
*F*_*eCA*_	Enhancement factor for eCA catalysis,	For *k*_+1_: $F_{eCA}^{*}\times \frac{10^{-8}}{\left[H^{+}\right]+10^{-8}}$	Steiner et al., 1975	/	/
		For *k*_−1_: $F_{eCA}^{*}\times \frac{\left[H^{+}\right]}{\left[H^{+}\right]+10^{-8}}$	Steiner et al., 1975		
*L*_*b*_	Effective thickness of diffusion boundary layer between bulk medium and the surface of the biomass	Estimated	/	20	μm
*D*_*O*_2__	Diffusion coefficient of dissolved oxygen	/	Wolf et al., 2007	2.00 × 10^−9^	m^2^·s^−1^
*D*_*C*_*O*__2__	Diffusion coefficient of dissolved free CO_2_	/	Wolf et al., 2007	1.91 × 10^−9^	m^2^·s^−1^
$D_{HCO_{3}^{-}}$	Diffusion coefficient of ${\text{HCO}}_{3}^{-}$ ion	/	Wolf et al., 2007	1.18 × 10^−9^	m^2^·s^−1^
$D_{CO_{3}^{2-}}$	Diffusion coefficient of ${\text{CO}}_{3}^{2-}$ ion	/	Wolf et al., 2007	9.14 × 10^−10^	m^2^·s^−1^
*D*_*Tris*_	Diffusion coefficient of Tris buffer	Estimated	/	1.0 × 10^−10^	m^2^·s^−1^
$D_{H^{+}}$	Diffusion coefficient of proton	/	Lee and Rasaiah, 2013	6.82 × 10^−9^	m^2^·s^−1^
$D_{OH^{-}}$	Diffusion coefficient of hydroxyl ion	/	Lee and Rasaiah, 2013	6.8 × 10^−9^	m^2^·s^−1^

^*^Refer to Table 2 and Figure 2; if not specified values shown here were used in the simulation.

** *A = 499002.24·e^4.2986× 10^−4^^·S^2^+5.75499 × 10^−5^·S*,

*R is the universal gas constant, equals 8.31451 J/mol*.

*** *Higher values indicate stronger eCA activity, a value of 1 indicates no eCA activity*.

## Results

### Profiles of dissolved oxygen, pH, and gross photosynthetic productivity

**Figure 3:**
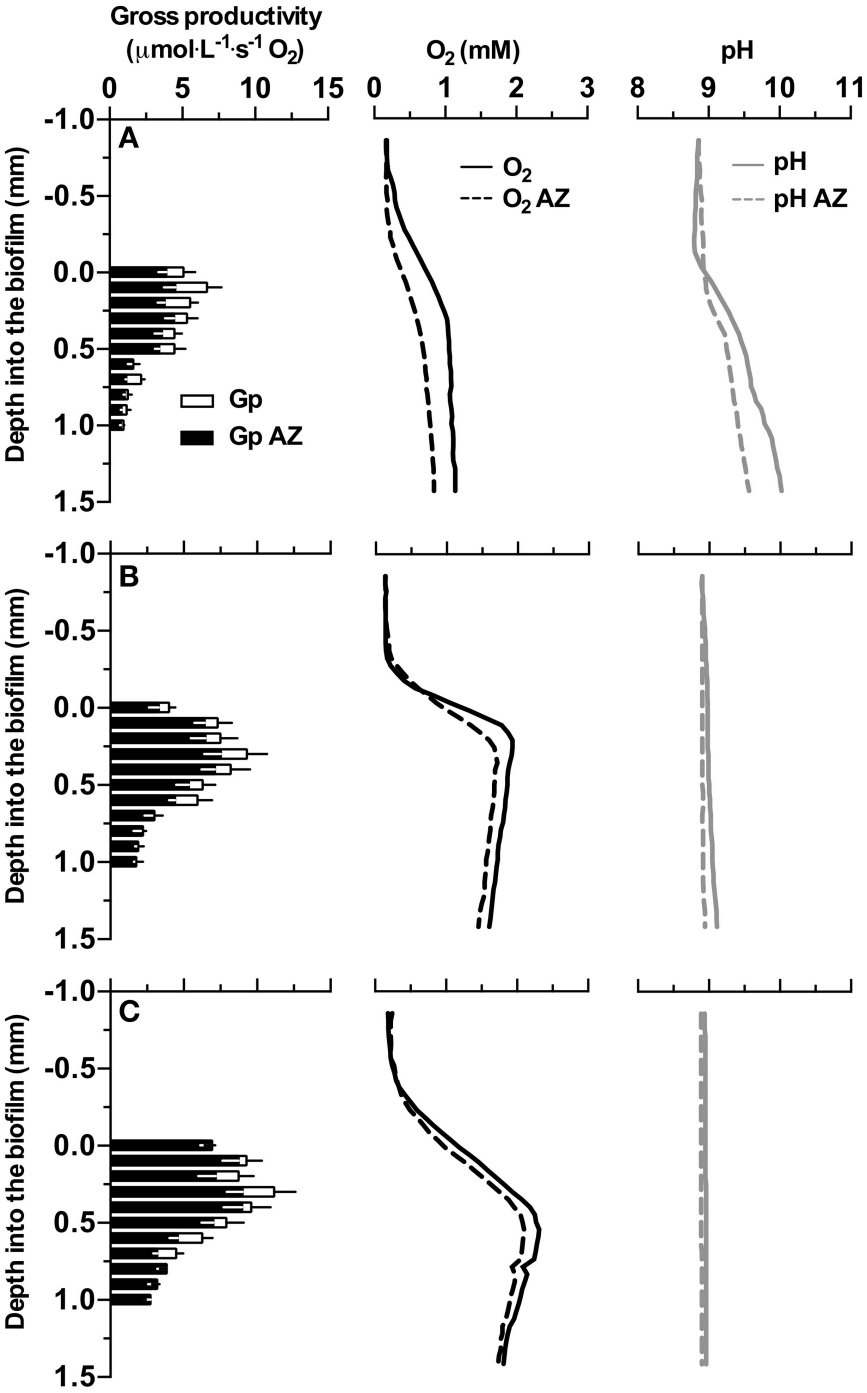
Profiles of gross photosynthetic activity (Gp), oxygen concentration, and pH measured with microsensors in the investigated biofilm. Row **(A–C)** give the profiles acquired from biofilms cultivated with medium supplemented with 0, 0.5, and 1 M dissolved inorganic carbon (DIC), respectively. Y-axes give the depth relative to the surface of the measured biofilm, 0 indicates the approximated position of the surface of the biofilm. Black bars represent Gp after AZ addition, empty bars show the difference of Gp before and after AZ addition. Solid lines represent profiles (i.e., O_2_, pH) before AZ addition, dashed lines represent profiles (i.e., O_2_, pH) after AZ addition. The data shown here are the mean values of 3 replicate measurements, for gross productivity, black and white error bars present the standard deviation for triplicates before and after AZ addition, respectively. Error bars for oxygen and pH measurements are omitted for clarity.

O_2_ production was observed in all samples under illumination (Figure 3). The thickness of the biofilm increased with higher DIC concentrations in the culture medium (visual observation) and also the O_2_ production increased with increasing DIC concentrations. A maximum O_2_ concentration of 2.3 mM was measured in biofilms cultivated with 1 M DIC. The pH increased from 8.7 at the surface to 10 in biofilms cultivated with low DIC (~13 mM) when eCA inhibitor was not present. In biofilms cultivated in media with high DIC (>0.5 M DIC), the pH increased less or not at all due to increased buffering of the carbonate system. After eCA inhibition, oxygen production decreased, especially at low DIC (oxygen flux was reduced from 1.7 to 1.3 ·μmole·m^−2^·s^−1^). The pH decreased only at low DIC, due to the low buffering strength of the medium. Biofilms cultivated at higher DIC concentrations suffered less productivity loss after eCA inhibition.

### Extracellular carbonic anhydrase and dissolved inorganic carbon uptake of resuspended biofilms

**Figure 4:**
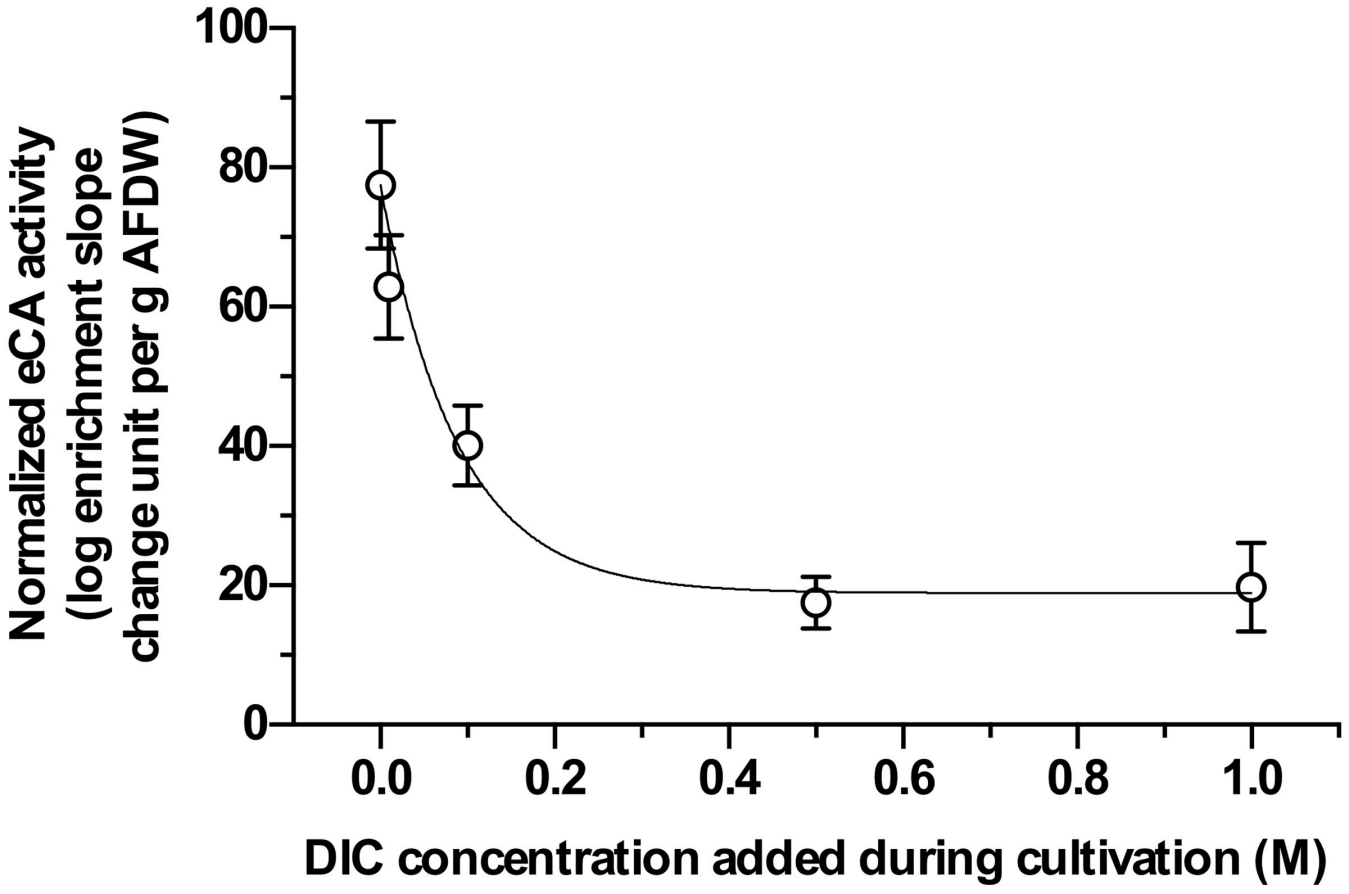
Extracellular carbonic anhydrase (eCA) activities of biomass cultivated with different dissolved inorganic carbon (DIC) concentrations, measured with membrane inlet mass spectrometry (MIMS). X-axis gives the supplemented DIC concentrations applied during cultivation. Y-axis shows the normalized eCA activity, calculated with Equation (2), normalized to per gram ash-free dry weight (AFDW). The data shown here are the mean values of 3 replicate measurements, error bars represent the standard deviations of the triplicates.

**Figure 5:**
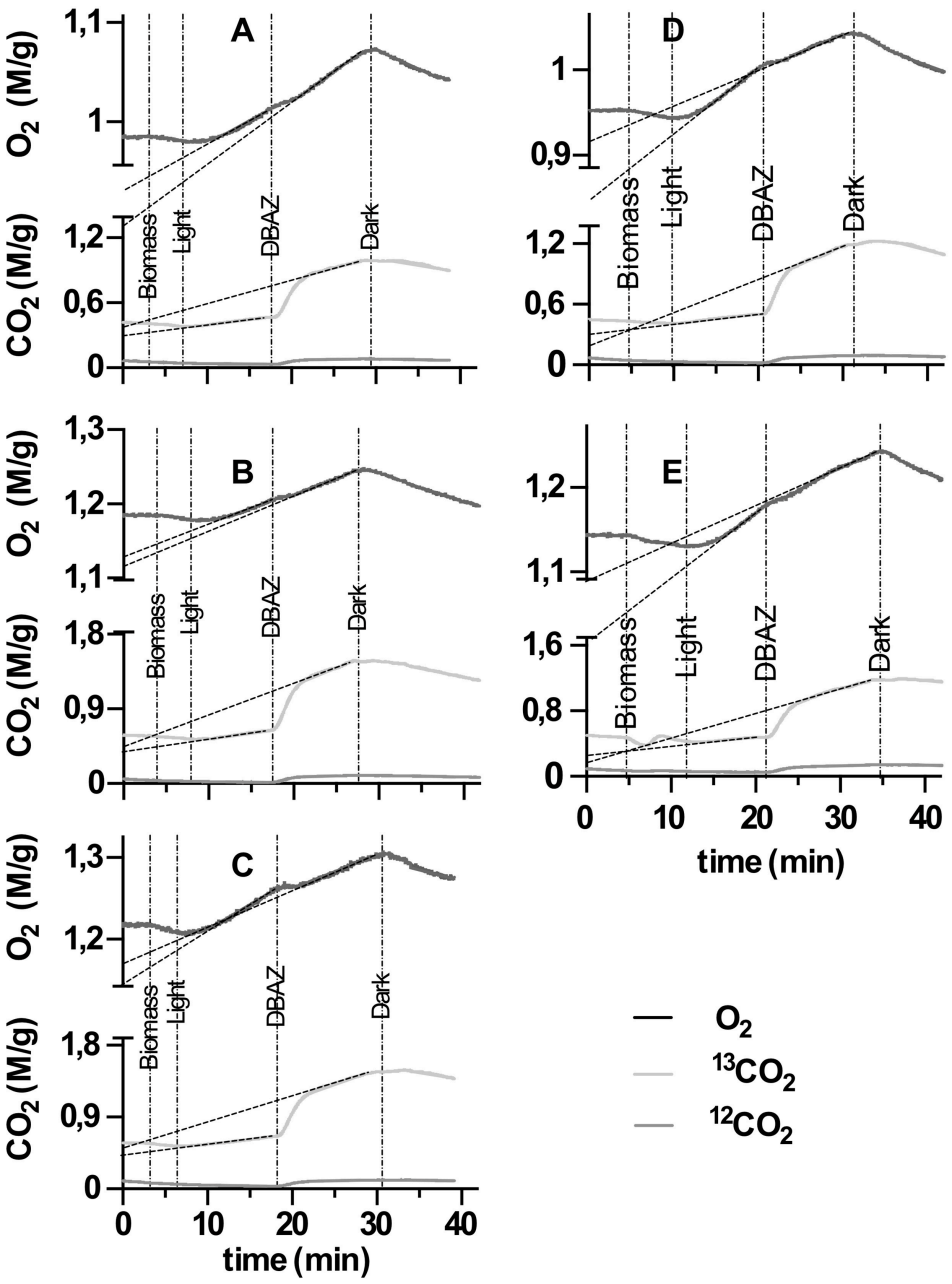
Time vs. concentration chart of membrane inlet mass spectrometry (MIMS) dissolved inorganic carbon (DIC) uptake experiments. Different panels show data acquired from biomass cultivated with different supplemented DIC concentrations: **(A**–**E)** represent biomass grown in 0, 0.01, 0.1, 0.5, and 1 M additional DIC, respectively. X-axis represent time. Y-axes are concentrations of O_2_ (top half-axis, black line) and ^13^CO_2_/^12^CO_2_ (bottom half-axis, lighter/darker gray lines) normalize through ash free dry weight of the biomass added during the measurement, respectively. The time point of events (injection of biomass, turn on light, injection of DBAZ, turn off light) are marked with thin vertical dotted-dashed lines and corresponding texts. The dashed lines present the regression lines of the relevant slopes in Figure 6.

**Figure 6:**
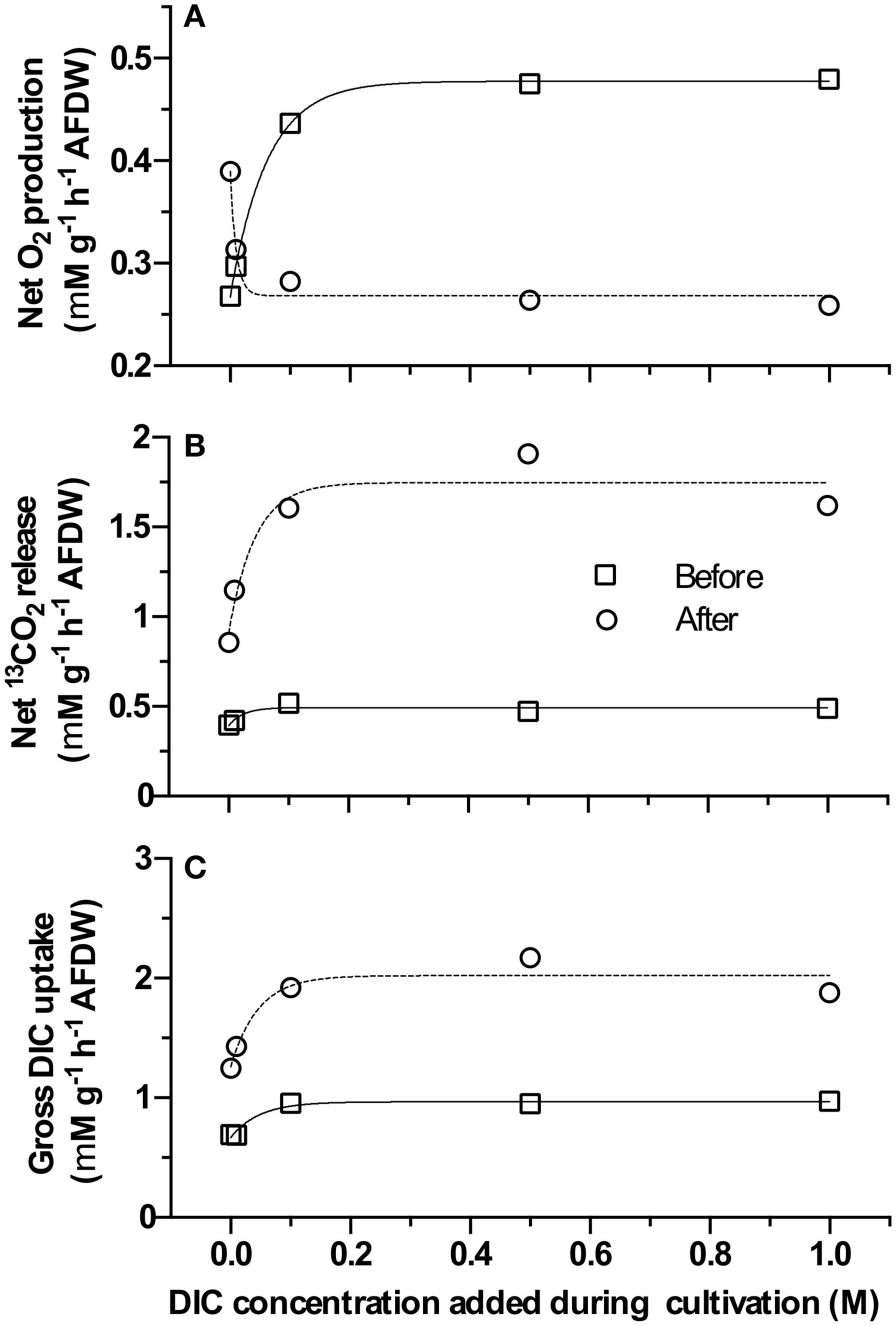
Net O_2_ production rate, ^13^CO_2_ release rate, and apparent gross dissolved inorganic carbon (DIC) uptake rate (calculated as the sum of Net O_2_ production rate and ^13^CO_2_ release rate) before/after DBAZ injection of the membrane inlet mass spectrometry (MIMS) DIC uptake experiments. X-axis gives the supplemented DIC concentrations applied during cultivation. Y-axes in **(A–C)** give the calculated absolute rates of net O_2_ production, ^13^CO_2_ release, and apparent gross DIC uptake rate, respectively (square and circle indicate rates before and after DBAZ injection).

All samples exhibited eCA activity, which decreased with increasing DIC concentration during cultivation. Above a DIC addition of 0.5 M no further decrease was observed. The effect of DIC addition on eCA activity could be four-fold (Figure 4). The dynamics of O_2_, ^13^CO_2_, and ^12^CO_2_ in response to illumination and eCA inhibition were similar for biomass grown at different DIC concentrations (Figure 5). Upon illumination, O_2_ started to increase, and, remarkably, ^13^CO_2_ also increased, indicating CO_2_ was released from the cells. Addition of DBAZ (an eCA inhibitor) induced a sudden release of ^13^CO_2_, after ~2.5 min, ^13^CO_2_ increased steadily again. The sudden release of ^13^CO_2_ is likely the combined result of unbinding of eCA bound ^13^CO_2_ due to addition of DBAZ, and the shift in equilibrium of the carbonate system caused by eCA inhibition (Palmqvist et al., 1994). Whereas, the steady increase is the leakage of ^13^CO_2_ into the bulk medium. DBAZ addition caused a decrease of O_2_ release for high DIC adapted biofilms (0.1, 0.5, and 1 M DIC) but, remarkably, accelerated O_2_ production in low DIC adapted biofilms. The increase in ^13^CO_2_ was steeper than when eCA was not inhibited, indicating an enhanced ^13^CO_2_ release and/or a slower ^13^CO_2_ depletion rate, e.g., conversion to bicarbonate, after eCA inhibition. After the light was turned off, O_2_ decreased immediately. ^13^CO_2_ continued to increase for a short period of time, after which the release of ^13^CO_2_ stopped. From data in Figure 5, oxygen production rates, ^13^CO_2_ release rates, and apparent gross DIC uptake rates (calculated as the sum of net oxygen production and ^13^CO_2_ release but does not account for the conversion of released ^13^CO_2_ into bicarbonate) were calculated (Figure 6). Without eCA inhibitor, net O_2_ production and ^13^CO_2_ release rates increased with supplemented DIC concentration during cultivation and leveled off at about 0.1 M DIC. The apparent gross DI^13^C uptake rates increased after eCA inhibition. Before inhibition of eCA, around 50% of the apparent gross DI^13^C uptake was released as ^13^CO_2_, whereas when eCA was inhibited, up to 87% of the apparent gross DI^13^C uptake was released as ^13^CO_2_.

### Modeling of DIC dynamics

**Figure 7:**
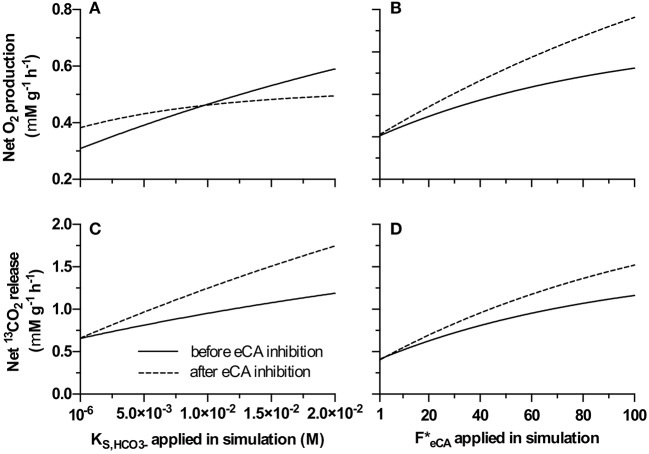
Net O_2_ production and net ^13^CO_2_ release before and after eCA inhibition (i.e. DBAZ injection), simulated using the proposed model. **(A,C)**: Analysis based on varying only $K_{S,HCO_{3}^{-}}$ value, **(A)** gives the net O_2_ production rate, **(C)** shows ^13^CO_2_ release rate; **(B,D)**: analysis based on varying only $F_{eCA}^{*}$ value, **(B)** gives the net O_2_ production rate, **(D)** shows ^13^CO_2_ release rate. In all panels, the solid lines give the rates before eCA inhibition; the dashed lines give the rates after eCA inhibition.

Similar to observed experimentally, the model suggested that, under certain conditions, net O_2_ production rate can be increased by inhibiting eCA (Figure 7). At lower $K_{S,HCO_{3}^{-}}$ values (<1 × 10^−2^ M·s^−1^, thus a high affinity to ${\text{HCO}}_{3}^{-}$) net oxygen production rate increased after the simulated eCA inhibition (Figure 7A). When other parameters were kept constant, and $K_{S,HCO_{3}^{-}}$ was low, the magnitude of this increase increased with higher *F*_*eCA*_ value (Figure 7B). Since the inhibition of eCA lead to higher ^13^CO_2_ release rates, and results in the accumulation of ^13^CO_2_ in the boundary layer, and lead to increased DIC availability. At higher $K_{S,HCO_{3}^{-}}$ values, after the simulated eCA inhibition, net oxygen production rate was decreased. For all tested $K_{S,HCO_{3}^{-}}$ values, net ^13^CO_2_ release rate increased after the simulated eCA inhibition, and the magnitude of this increase was stronger with higher $K_{S,HCO_{3}^{-}}\;$values.

## Discussion

Previously, eCA has been shown to participate in DIC uptake during microalgal photosynthesis, and eCA has been detected in the mat forming cyanobacterium *M. chthonoplastes* (Kupriyanova et al., 2003, 2007, 2011). In the present study, an inhibitor of eCA, acetazolamide (AZ) was applied to the biofilm during the microsensor measurement. Inhibition of eCA led to decreases in both O_2_ concentrations in the biofilm and gross photosynthetic productivities, especially for low DIC (~13 mM) cultivated biofilms. This shows directly the presence of eCA and its positive effect on photosynthetic oxygen production. The activity of eCA was further verified by the MIMS measurements (Figure 4). Previous studies investigating the role of eCA in DIC uptake mainly focused on planktonic microalgae, exposed to much lower DIC concentrations and lower pH values than in the present study (Palmqvist et al., 1994; Nimer et al., 1999; Rost et al., 2007; Beckmann et al., 2009; Kupriyanova et al., 2011; Hamizah et al., 2017). Our results confirmed the activity of eCA and showed its participation in bicarbonate uptake by biofilm-inhabiting cyanobacteria.

The fact that the activity of eCA is downregulated at increasing DIC levels, further demonstrates its role in DIC uptake. The function of eCA is to accelerate the extracellular interconversion from CO_2_ to bicarbonate, i.e., to a DIC species that can be actively taken up by the cells. As a result, DIC uptake is enhanced and subjected to regulation by the cells (Giordano et al., 2005). Most importantly, eCA prevents leaked out CO_2_ from escaping from periplasmic space into the bulk liquid. Most of the CO_2_ reaching the periplasm originates from inside the cells, where it is produced from ${\text{HCO}}_{3}^{-}$. The microsensor measurements showed that, in biofilm cultivated with no additional DIC, the pH decreased after eCA inhibition (Figure 3), indicating a lower rate of CO_2_ fixation.

Both CO_2_ and ${\text{HCO}}_{3}^{-}$ can serve as extracellular carbon sources, whereby CO_2_ is taken up passively by diffusion and bicarbonate by active transport. CO_2_ uptake does not cost energy (i.e., passive diffusion), but cannot drive accumulation of DIC in the cell (Giordano et al., 2005). Active ${\text{HCO}}_{3}^{-}$ uptake requires energy supplied by photosynthesis (Giordano et al., 2005). Cyanobacteria have 3 known active ${\text{HCO}}_{3}^{-}$ uptake pathways for accumulation of DIC inside their cells (Omata et al., 1999; Shibata et al., 2002; Price et al., 2004, 2008; Raven et al., 2008). Under the experimental conditions in this study (i.e., pH buffered to 8.7) the intracellular DIC level will be higher than that in the medium, as cyanobacteria cells can concentrate DIC intracellularly up to 1,000-fold the extracellular level. If in the cytoplasm the pH is 7.3, the CO_2_ concentration in the cytoplasm is likely higher than that in the bulk medium as a result of a much higher total DIC concentration and a lower pH, even if the carbonate system is not in equilibrium. In the cyanobacterial cells, ${\text{HCO}}_{3}^{-}$ is transported to the carboxysomes, and converted to CO_2_ for RuBisCo consumption, thus the CO_2_ concentration inside the carboxysomes can be further elevated (Price et al., 2008; Raven et al., 2008; Kerfeld and Melnicki, 2016). These CO_2_ concentration gradients lead to the observed ^13^CO_2_ release under illumination during the MIMS measurement.

In suspension (i.e., during MIMS measurement) inhibition of eCA leads initially to a steep increase in ^13^CO_2_ release. After this initial increase, increases in the steady state ^13^CO_2_ release rates was also observed (Figure 6). This shows that eCA reduces the CO_2_ leakage into the bulk medium. Interestingly, the apparent gross DIC uptake rates were increased markedly after eCA inhibition in the experimental MIMS measurement (Figure 6C). A similar trend was also predicted by the proposed model (Figure 7). This suggests that the enhanced CO_2_ leakage could be compensated by a higher ${\text{HCO}}_{3}^{-}$ uptake, either by promoting active ${\text{HCO}}_{3}^{-}$ uptake or by increasing ${\text{HCO}}_{3}^{-}$ availability. Figure 6 shows, when eCA was active, 50% of the DIC uptake was assimilated or converted to bicarbonate for reuptake, the remainder escaped by CO_2_ leakage into the bulk medium. When eCA was inhibited, for higher DIC cultivated biomass, only <13% of the DIC uptake could be assimilated or converted to bicarbonate, while the rest escapes into the bulk medium.

The positive effect of eCA on DIC uptake could also be observed in the biofilm, especially for the biofilm cultivated at low DIC. For high DIC biofilms, the effect was much less pronounced. Mass transfer in biofilms is limited by diffusion, leading to significant mass transfer resistance. Such a resistance does not exist in suspensions (Dibdin, 1997). Consequently, CO_2_ that has leaked out of cells, is largely retained in the biofilms and not lost into the medium. It thus remains available for re-uptake. In this scenario, where generated intermediates constantly leak out of cells, mass transfer resistance can enhance the uptake of substrate. At low DIC concentration (~0.13 mM DIC), DIC can be limiting inside the biofilm, making the eCA more important. At higher DIC concentrations, DIC becomes more readily available inside the biofilm, thus the eCA activity was less important (Figure 4).

Thus, the function of eCA is probably the promotion of DIC assimilation (i.e., photosynthetic carbon fixation efficiency). Remarkably, in low DIC cultivated biomass, inhibition of eCA induced a stimulation of the photosynthesis rate in suspensions (Figure 6A). To better understand this observation, an analysis of the system was performed using the dynamic model. Microalgae adapt to low bicarbonate concentrations by increasing their affinity for bicarbonate (i.e., decrease the half saturation concentrations for bicarbonate uptake), that also reduces the maximum uptake- and assimilation rate of bicarbonate (Aizawa and Miyachi, 1986; Raven et al., 2008). A biofilm that is adapted to low bicarbonate concentrations might exhibit only a slight increase in photosynthetic rate at high bicarbonate concentrations, as it is limited by lower maximum rates. Conversely, a biofilm adapted to high bicarbonate concentrations will become bicarbonate limited at low bicarbonate concentration.

With this concept in mind, the remarkable increase in O_2_ production after eCA inhibition for low DIC cultivated biomass (Figure 6A) can be explained. After transferring the biomass to a higher DIC concentration, the bicarbonate uptake is limited by the maximum uptake rate (e.g., limited by the number of bicarbonate transporters). As eCA converts CO_2_ to bicarbonate, its inhibition increased the concentration of dissolved free ^13^CO_2_ in the cell boundary (e.g., the periplasm) that always can pass the membranes. Since this biomass has low maximum uptake rate for ${\text{HCO}}_{3}^{-}$, the ${\text{HCO}}_{3}^{-}$ uptake is over-saturated when the biomass was transferred to higher DIC medium (13 mM during cultivation and 0.1 M DIC during the MIMS measurement). In this special case eCA inhibition increased the CO_2_ concentration in the biomass without affecting ${\text{HCO}}_{3}^{-}$ uptake, leading to higher O_2_ production rate. Too further clarify, the MIMS measurements were performed in comparatively much higher DIC concentration for biomass cultivated at low DICs. Our hypothesis is that this biomass has adapted to the low DIC environment, i.e., the ${\text{HCO}}_{3}^{-}$ transport of these biomass have high affinity to ${\text{HCO}}_{3}^{-}$ but low maximum ${\text{HCO}}_{3}^{-}$ uptake rate. This means during the MIMS measurement, a decrease in periplasmic ${\text{HCO}}_{3}^{-}$ concentration can still support the same ${\text{HCO}}_{3}^{-}$ uptake rate as before eCA inhibition, as the ${\text{HCO}}_{3}^{-}$ concentration is still high enough for low DIC adapted biomass to maintain close to maximum uptake rate (as before eCA inhibition). However, a higher CO_2_ concentration due to a slower conversion rate to ${\text{HCO}}_{3}^{-}$ in the periplasmic space increased CO_2_ concentration in the periplasmic space, and thus slowed the leakage of CO_2_, making CO_2_ more available inside the cells for assimilation. For high DIC adapted biomass, at 0.1 M DIC, the DIC uptake is limited by the lower concentration rather than the maximum uptake rate, i.e., under-saturated. Inhibition of eCA decrease the availability of bicarbonate, thus causing the net productivity to decrease. The observed increase of photosynthesis upon inhibiting eCA, in this special case, was indeed an outcome of the model (Figure 7).

## Conclusion

To summarize, eCA converts CO_2_ escaping from the cytoplasm into the periplasmic space into bicarbonate, which can be taken up again by the cell. In suspensions, eCA reduced the CO_2_ leakage to the bulk medium from 90 to 50%. In biofilms cultivated with low DIC, the oxygen production was reduced by more than 25% upon eCA inhibition. The role of eCA in biofilms was much less significant at high DIC (0.5–1 M). Despite a stronger eCA activity, lower DIC adapted biomass exhibited lower net productivity and lower apparent gross DIC uptake rate. Both eCA production and bicarbonate uptake consumes energy, with the former dependent on the amount of eCA produced/maintained, and the latter dependent on the amount of uptake and the concentration of bicarbonate. Consequently, it can be suspected: the biofilm adapts to high DIC concentrations by decreasing the activity of eCA and increasing the DIC uptake rate.

To further verify this hypothesis, future studies on the DIC uptake kinetic (e.g., MIMS DIC uptake measurement with a range of DIC concentrations) should be performed. In the present study, all MIMS measurements were performed using biomass homogenized across the depth of the entire biofilm. It is possible, due to gradients (e.g., pH, light) in the investigated biofilms, biomass at different depths of the biofilm have different eCA activities and/or DIC uptake related parameters. Thus, similar measurements on biomass acquired from different depths of the biofilm (e.g., by sectioning the biofilm) should also be carried out in future studies.

## Author contributions

TL performed most of the experimental, theoretical work, and the writing. CS and MA performed part of the experiment. All authors took part in the discussion of the data and participated in the writing of the manuscript.

### Conflict of interest statement

The authors declare that the research was conducted in the absence of any commercial or financial relationships that could be construed as a potential conflict of interest.
